# Influence of Lifestyle Factors on Oral Health-Related Quality of Life in Pregnant Women in Indore City

**DOI:** 10.21315/mjms2018.25.2.13

**Published:** 2018-04-27

**Authors:** Sandeep Kumar, Bhumika K Badiyani, Afsheen Lalani, Amit Kumar, Sayak Roy

**Affiliations:** 1 Department of Public Health Dentistry, Dental Institute, RIMS, Ranchi, Jharkhand, India; 2 Department of Public Health Dentistry, Sarjug Dental College and Hospital, Darbhanga, Bihar, India; 3 Department of Public Health Dentistry, Sri Aurobindo College of Dentistry, Indore, Madhya Pradesh, India; 4 Department of Oral Medicine and Radiology, Dafodyl Dental Clinic, Kolkata, West Bengal, India

**Keywords:** pregnant women, lifestyle factors, oral health-related quality of life

## Abstract

**Background:**

Lifestyle factors affect the periodontal and oral hygiene status and, thus, may affect the Oral Health-Related Quality of Life (OHRQoL) in pregnant women. Thus, the aim of the study was to assess the OHRQoL and determine its relationship with lifestyle and other factors in pregnant women in Indore city.

**Methods:**

This cross-sectional study was carried out on 400 pregnant women who were selected using stratified random sampling technique from eight private maternity centers located in Indore city. A questionnaire collected information on socio-demographic characteristics, oral hygiene practices, previous dental visit and past medical history. OHRQ_O_L was assessed using Oral Health Impact Profile-14 questionnaire. Lifestyle factors were assessed using the Health practice Index.

**Results:**

The lifestyle factors were the strongest predictor for poor OHRQ_O_L. The pregnant women (OR = 3.22, *P*-value < 0.0001*) with poor lifestyle had significantly poor OHRQ_O_L. Logistic regression analysis showed that poor socio-economic status (OR = 2.63, *P*-value = 0.025*), brushing frequency of less than or equal to once daily (OR = 2.02, *P*-value = 0.025*), and suffering from systemic diseases (OR = 2.11, *P*-value = 0.017*) were other important predictors for poor OHRQ_O_L in pregnant women.

**Conclusions:**

Our findings showed that lifestyle factors significantly impact OHRQ_O_L in pregnant women. Thus, it is recommended that effective policies should be drafted to improve lifestyle factors and OHRQ_O_L in pregnant women.

## Introduction

Pregnancy is usually considered as a moment of happiness for expecting mothers (1). However, it is associated with many dental and oral problems that might affect the Oral Health-Related Quality of Life (OHRQoL). These problems include dental caries, erosion, pregnancy-induced gingivitis, periodontal infections, pregnancy epulis, increased tooth mobility, and dental problems related to labor and delivery (2, 3, 4).

With increased levels of principal female sex hormones, viz., estrogen and progesterone, significant physiological changes are seen in expecting mothers due to the synergistic effect of these hormones in controlling the menstrual cycle, maintenance of pregnancy, and the initiation of labor. It has been observed that, compared to the levels seen during the menstrual cycle, the estrogen and progesterone levels may reach 30 times and 10 times higher, respectively, during pregnancy. The increase in the levels of these hormones can significantly affect the major organ systems, including the periodontium (4, 5). These increased hormonal levels cause increased blood supply to the gingival tissue, resulting in swollen and bleeding gums known as gingivitis (6). The tooth mobility reported during pregnancy is mostly due to diseases affecting the periodontal ligaments. It should be noted here that pregnancy can only modify other conditions that can result in dental diseases.

Moreover, lifestyle factors also have a major role in oral health and behaviour (7). Pregnant women undergo several changes in their lifestyle that may affect their oral health and OHRQoL. Although there are many studies on OHRQoL (1, 8) or lifestyle factors (7, 9) in pregnant women, the impact of lifestyle factors on OHRQoL has not been assessed so far. Therefore, the aim of this study was to examine OHRQoL and its relationship with lifestyle and other factors among pregnant women in Indore city, India.

## Materials and Methods

This cross-sectional study included pregnant women, aged 20–45 years, selected from eight Private Maternity Centers in Indore City, India, using stratified random sampling method and was conducted for three months (July–September 2016). For the selection of study population, Indore city was divided into four zones; two maternity centres were randomly selected from each zone. The ethical approval to conduct the study was taken from Sri Aurobindo Institute of Medical Sciences with ethical clearance number IERC/2013/No 7. Based on the findings of a pilot study, the final sample size was calculated for this study. The intended sample size was calculated to be 374, keeping the power of study at 80% and alpha error at 5% with anticipated 55% prevalence of at least one impact on OHRQoL. It was rounded off to 400 pregnant women in order to compensate for non-response. Statistical software N Master 2 (CMC, Vellore) was used to calculate the sample size. A questionnaire was prepared to collect information on socio-demographic characteristics, past medical history, oral hygiene practices, and previous dental visits of the participants.

The Oral Health Impact Profile-14 (OHIP- 14) questionnaire was used to assess OHRQoL (10). It consisted of 14 items organised in seven domains (functional limitation; physical pain; psychological discomfort; physical disability; psychological disability; social disability; and handicap). The responses were categorised on a five-point Likert scale (1. very often; 2. fairly often; 3. occasionally; 4. hardly ever; 5. never). The questionnaire intended to determine whether the pregnant women had any difficulty in the seven domains in the last twelve months. The minimum score was 0, and the maximum score was 56. A higher score indicated poor OHRQoL. For the sake of convenience, OHRQoL was dichotomised at its median value, and a score ≥ 28 indicated poor OHRQoL.

The Health Practice Index (HPI) was used to assess different lifestyle factors. This index consisted of an eight-item scale, ranging 0–8. The questionnaire collected information regarding smoking, consumption of alcohol, eating breakfast, hours of sleep/night, hours of work/day, physical exercise, nutritional balance, and mental stress. The “good” health practices were coded 1, and “poor” health practices were coded as 0. Based on the number of good health practices, each subject was assigned a score between 0 and 8 and classified into the following three categories based on the Morimoto criteria: poor lifestyle (score = 0–3), moderate lifestyle (scores = 4–5), and good lifestyle (scores = 6 or higher) (11, 12).

The English version of HPI was translated into the Hindi language. Its validity was checked using the back translation method, involving blind re-translation into English and verified by experts in both languages. This index was also checked after wording modification to ensure the functional and conceptual equivalence of the questionnaire.

The socio-economic status of the participants was calculated using the modified Kuppuswamy scale. Based on their status, the individuals were broadly classified into three major categories: upper class, middle class and lower class (13).

The pregnant ladies who were intellectually and physically capable of responding, willing to participate, and signed the informed consent were included in the study. Those with active habits of drinking alcohol and smoking, using prophylactic antibiotics due to any reason, or undergoing periodontal treatment during the past six months were excluded from the study. The included pregnant ladies were asked to fill the self-administered pro forma under the supervision of single trained investigator. The response rate was 100%. Any doubts arising during the filling of the pro forma were clarified by the investigator.

All the collected data were analysed using SPSS (version 20). Descriptive and analytical tests were performed. Bivariate analysis followed by logistic regression analysis [ENTER Method] were also performed. *P*-value < 0.05 was considered statistically significant.

## Results

More than 50% of the pregnant women were in the age group of above 30 years, belonging to lower class, and resided in urban areas. A majority of the pregnant women used toothbrush and toothpaste and no mouthwash for maintenance of oral hygiene. Nearly 85% of the pregnant women did not suffer from any systemic disease or reported previous pregnancies. In addition, most of the participants had never visited any dentist for any problems related to oral health. The HPI Index showed that nearly half of the pregnant women had poor lifestyle score.

The results of the OHIP-14 questionnaire showed that nearly one-third of the pregnant women had poor OHRQoL (Table 1). A bivariate analysis using frequency distribution and Chi-square test was performed to identify the factors associated with poor OHRQoL. The identified factors were socio-economic status, frequency of brushing, individuals suffering from any systemic diseases and lifestyle scores (Table 2).

The significant factors in bivariate analysis were then entered into multivariate analysis to identify the strength of association. As mentioned earlier, the OHRQoL was dichotomised at its median value, and the scores ≥ 28 indicated poor OHRQoL, which was kept as a dependent variable. The socio-economic status, frequency of brushing, individuals suffering from any systemic diseases, and lifestyle score were considered as independent variables. The pregnant ladies belonging to lower socioeconomic status (OR = 2.63, *P*-value = 0.025*) and brushing less than or equal to once daily (OR = 2.02, *P*-value = 0.025*) showed poor OHRQoL. Those suffering from some systemic diseases (OR = 2.11, *P*-value = 0.017*) were more likely to have poor OHRQoL. The lifestyle factors were found to be the strongest predictor for poor OHRQoL. Those with a poor lifestyle score (OR = 3.22, *P*-value < 0.0001*) showed poor OHRQoL. The Model fitting criteria showed M log R value to be 98.286, which is suggestive of a good fit (Table 3).

## Discussion

This study assessed OHRQoL and determined its relationship with lifestyle and other factors in pregnant women in Indore city. In the present study, nearly 30% of the included pregnant women were found to have poor OHRQoL. This finding is in accordance with the findings of previous studies carried out by Acharya et al. (1) and Pramila et al. (14). Amar and Chung (4) reported that pregnant women suffer from hormonal imbalance, which can lead to poor OHRQoL during pregnancy.

Factors like gender, behavior, general health, education, income, smoking, alcohol consumption, dietary habits, behavior of parents, physical and social activity have been reported to be associated with dental health behavior (15–18). The study of lifestyle as a factor helps to examine behavior in a wider sense and sheds more light on the personal characteristics of an individual compare to previous investigations that included only dental health habits or smoking habits (19). In a review done by Homan et al. (20), lifestyle factors were found to have a profound impact on reproductive health. In the present study, 49% of the pregnant women had poor lifestyle as assessed using the HPI scores. Similar findings have also been reported by Santiago et al. (21). It was also found that pregnant women with a poor lifestyle score were more likely to have poor OHRQoL.

In our study, those pregnant women belonging to lower socio-economic status had poor OHRQoL. Several other studies have also found similar socio-economic inequalities with OHRQoL (22–25). It has also been reported that the lack of dental awareness, the lack of utilisation of dental services in socially deprived areas, and social and environmental factors are the main factors responsible for such inequalities.

The pregnant ladies who brushed their teeth less frequently were reported to have poor OHRQoL than individuals with a higher frequency of brushing (≥ twice/daily). Similar findings were reported by Aimée et al. (26) and Jamieson et al. (25). Brushing the teeth twice a day helped in better plaque control, leading to better oral hygiene maintenance and OHRQoL.

The individuals suffering from systemic diseases were reported to have poor OHRQoL. Similar findings were reported by Rebelo et al. (22) and Busato et al. (27).

## Limitations

This study has certain limitations. First, this study had a cross-sectional design. The pregnant women were selected from private maternity centers since taking permissions from Government institutions involved a long and complicated process that was very tedious and time taking. It should be noted here that the objective of the study was not affected because pregnant ladies from all sections of society (rural and urban) visited private maternity centers. However, the authors felt it necessary to mention this as a limitation of the study. Moreover, there may be other factors responsible for poor OHRQoL in pregnant ladies that might not have been taken into consideration. Hence, further longitudinal studies using a larger sample size and equal representation from private and government-run institutions need to be carried out before the results can be generalised.

## Conclusion

Our findings showed that lifestyle factors play an important role in OHRQoL. The socioeconomic status, brushing frequency and systemic diseases are other factors that affect OHRQoL. Thus, it is recommended that dental health education should be provided and that effective policies should be drafted and implemented to improve the lifestyle factors and OHRQoL in pregnant women.

## Figures and Tables

**Table 1:** Distribution of study population based upon oral health related quality of life (OHRQoL)

Categories	*N* (%)
Poor	120 (30.0%)
Good	280 (70.0%)

**Table 2:** Frequency distribution and bivariate analysis to identify the factors associated with poor OHRQoL (Score ≥ 28)

Factor	Categories	Frequency distribution *N* (%)	Unadjusted Odds ratio	95 % CI	*P*-value
Age	≤ 30 years	171 (42.8%)	1.09	0.71, 1.67	0.708
	> 30 years	229 (57.2%)	1.00		
Socioeconomic status	Upper class	48 (12.0%)	1.00		
	Middle class	134 (33.5%)	1.53	0.96, 2.50	0.073
	Lower class	218 (54.5%)	2.70	1.22, 6.25	0.015*
Location	Urban	237 (59.3%)	1.09	0.71, 1.70	0.673
	Rural	163 (40.8%)	1.00		
Oral hygiene aid	Toothbrush	268 (67.0%)	1.11	0.62, 1.97	0.732
	Finger	60 (15.0%)	1.30	0.62, 2.74	0.490
	Other	72 (18.0%)	1.00		
Material used	Toothpaste	333 (83.3%)	1.12	0.39, 3.21	0.839
	Toothpowder	49 (12.3%)	1.15	0.35, 3.79	0.822
	Other	18 (4.5%)	1.00		
Frequency of brushing	≤ Once daily	316 (79.0%)	2.09	1.15, 3.77	0.015*
	Twice or more daily	84 (21.0%)	1.00		
Technique of brushing	Vertical	70 (17.5%)	1.04	0.52, 2.11	0.908
	Horizontal	70 (17.5%)	0.91	0.45, 1.86	0.802
	Combination	188 (47.0%)	0.84	0.46, 1.51	0.552
	Other	72 (18.0%)	1.00		
Mouthwash use	Yes	34 (8.5%)	0.47	0.19, 1.18	0.107
	No	366(91.5%)	1.00		
Systemic disease	Yes	62 (15.5%)	1.88	1.07, 3.28	0.027*
	No	338 (84.5%)	1.00		
Previous pregnancy	Yes	60 (15.0%)	0.83	0.45, 1.53	0.542
	No	340 (85.0%)	1.00		
Dental visit	Yes	70 (17.5%)	1.18	0.68, 2.04	0.566
	No	330 (82.5%)	1.00		
HPI Total	Good	109 (27.3%)	1.00		
	Fair	95 (23.8%)	0.38	1.49, 4.76	0.001*
	Poor	196 (49.0%)	3.33	1.88, 5.88	< 0.0001*

* *P-*value < 0.05: statistical significant difference

**Table 3:** Multivariate analysis to identify the predictors with poor OHRQoL (score ≥ 28)

Factor	Categories	Adjusted Odds ratio	95 % CI	*P*-value
Socioeconomic status	Upper class	1.00		
	Middle class	1.96	1.16, 3.22	0.011*
	Lower class	2.63	1.14, 6.25	0.025*
Frequency of brushing	≤ Once daily	2.02	1.09, 3.73	0.025*
	Twice or more daily			
Systemic disease	Yes	2.11	1.15, 3.89	0.017*
	No			
HPI Total	Good	1.00	0.18, 0.56	
	Fair	2.63	1.47, 4.76	0.001*
	Poor	3.22	1.78, 5.55	< 0.0001*

* *P*-value < 0.05: statistical significant difference
